# Long-Term Outcome of Adolescent Anorexia Nervosa: Family Treatment Apartments Compared With Child Psychiatric Inpatient Treatment

**DOI:** 10.3389/fpsyt.2021.640622

**Published:** 2021-05-17

**Authors:** Ulf Wallin, Riitta Holmer

**Affiliations:** Centre of Eating Disorders, Psychiatry Skåne, Lund, Sweden

**Keywords:** anorexia nervosa, children and adolescence, follow- up study, family based treatment, in patient hospital treatment

## Abstract

**Introduction:** The family is rarely involved in treatment when the patient with anorexia nervosa (AN) is hospitalized. Family treatment apartment (FTA) represents an intervention that includes the family in the intensive treatment of AN. This study compares the short- and long-term outcomes of adolescents treated in FTA with those who received inpatient hospital care. In FTA, the parents are responsible for providing meal support, whereas in hospital care, the staff is responsible.

**Methods:** Sixty-eight previous patients admitted during the period 1990–2009 participated in a follow-up, 43 from the FTA where the whole family is admitted for treatment and 25 from regular psychiatric inpatient care. The follow-up consisted of a personal meeting with structured interviews, measurement of height and weight, and self-rating questionnaires.

**Result:** Readmissions due to weight loss within 6 months from discharge were less common in the FTA group. At follow-up, 14.2 years after admission, there was no difference in eating disorder pathology between the groups. There were significantly lower scores on general psychiatric pathology and significantly higher scores on quality of life in the FTA group.

**Discussion:** The treatment in FTA aims to give the family the ability to handle AN when it is most challenging. FTA may thus provide a helpful context for treatment with a basic sense of security along with skills that could contribute to better general mental health at follow-up.

## Introduction

When patients are seriously ill and in need of inpatient care, it is often the case that the family is not involved, and the family treatment ceases. It is well-established in treatment research that the family is a central resource for the young patient with anorexia nervosa (AN) to be able to recover, and family-based treatment (FBT) has demonstrated its superiority in repeated studies (1). These studies have mainly been conducted in outpatient care and with recent-onset cases. Knowing how to involve the family in a higher level of care is scarce, and the need to improve research has been underlined in a recent meta-analysis (2).

At the Center of Eating Disorders in Lund, Sweden, the family treatment apartment (FTA) model was developed in 1990. In FTA, the whole family is admitted for 5–6 weeks, and the treatment is based on the family therapy developed at the Maudsley hospital in London (3). FTA was developed as a high-intensity FBT serving as an alternative to psychiatric inpatient care when the patient was in such poor physical and psychiatric health that outpatient care was no longer sufficient.

Family-based programs in a higher level of care have recently come into focus for development. Descriptions of treatment programs involving families in a higher level of care have been published (4–6). Five recently published studies try to evaluate the effect of implementing family treatment in Family-Based Partial Hospitalization Programs (7–11). They all evaluate programs that engage parents in taking responsibility for the patient's food intake. One of the studies (7) has no follow-up, the other four have a 3- or 6-month follow-up. They all describe improvement during treatment, which is maintained at follow-up.

The use of family therapy for inpatients is not well-researched. We found five studies that examine inpatient programs that incorporate families to support the child with meals and the recovery process (12–16). The different treatment programs are similar in that the parents take responsibility for the meals. One difference between the program is the treatment setting. The treatment is usually integrated into the inpatient unit with the other patients, but in some programs, such as in FTA, the family has its own apartment.

One study from the eating disorders unit in Sydney (4, 12) describes a treatment model similar to the FTA model. The adolescent and his/her family had a 2-week family-based hospital admission at the outset of hospital treatment. In Fink et al. (12), the authors conclude that this treatment program provides struggling families with enhanced skills and a stronger foundation for outpatient FBT.

There are two Scandinavian studies on how to integrate family therapy components into the treatment of AN at inpatient units. The first study (13) is from the Regional Section of Eating Disorders (RASP) in Oslo and is a follow-up study after 4.5 years of 37 patients. One of the parents was present at the unit at all times; in two-thirds of the cases, both parents stayed at the unit initially. Siblings were also welcome, but in most cases, they stayed at home. The family treatment aimed to help parents establish clear, predictable frameworks for meals.

The second Scandinavian study (16) compared the family inpatient unit at Stockholm's Center for Eating Disorders (SCÄ) with an inpatient unit at an eating disorder unit in Copenhagen. The inpatient unit in Copenhagen did not include the family. They found shorter hospital stays and fewer readmissions at the family unit, which may indicate that when the family focuses on the treatment, the result is more durable. It can be difficult to assess the significance of this finding, as there may be different guidelines and traditions regarding criteria for admission and length of admissions in the two different countries.

In Matthews et al. (14), the patient and the family received an FBT intervention while the patient was hospitalized for medical complications of AN. The components of FBT (e.g., psychoeducation, illness externalization, minimizing guilt, and blame) were coupled with intensive caregiver meal coaching and parent-directed behavioral contracting. The authors compared the intervention group with a retrospective treatment-as-usual group at 3 and 6 months after discharge. The group that received FBT intervention gained significantly more weight.

In another study (15), the parents were asked to be present as much as possible throughout the admission. Each patient's family was provided with FBT adapted for an inpatient setting for the duration of the admission. Parents were encouraged to provide support for all meals in the hospital and to plan for meals out of the hospital. This study demonstrated the feasibility of implementing FBT principles in an inpatient program.

All studies described improvement, but only two studies (14, 16) had a comparison group. Matthews et al. (14) compared with patients who had been treated before starting family therapy, and Fjelkegård et al. (16) compared with patients from inpatient care at another unit. Both studies conclude that the outcome of treatment seems more sustainable when the family is involved.

Although, we know that the long-term course for adolescent AN is protracted, it is not clear whether the family influences the illness course in the long term. Rydberg Dobrescu et al. (17) showed in their 30-year follow-up of a community-based sample that one in five had a chronic eating disorder, whereas, 64% were fully recovered. The long-term course for hospitalized children and adolescents may be worse compared with those who were followed up in the Rydberg Dobrescu study. The long-time follow-up studies have varying results (18–22). In these studies, the follow-up time varied (a mean value of 7.5–20 years). The age of the patients when admitted to treatment varied (between 9 and 22 years). The proportion of participants reaching full recovery varied between 41% (after 7.5 years) (21) and 75.8% (after 12 years) (18).

The risk for relapse and the need for rehospitalization are high during the first year after discharge. Andrés-Pepiñá et al. (22) followed up the participants for 12 months after discharge and found that 24.8% required readmission after complete weight recovery.

## Aim Of The Study

The study aims to investigate whether the long-term course differs between those who have been in FTA compared with those who have been in traditional inpatient treatment. We also wanted to investigate if the short-time course is affected if the family has been involved in the treatment when the patient was very sick. This retrospective cohort study aims to investigate the long-time and short-term course for patients who had been in FTA with patients with AN who had been treated during the same period in inpatient care at the Child and Adolescent Mental Health Service (CAMHS) in Malmö.

Research has shown that FBT has a superior effect on the short-term course, but the effect on the long-term course had not been demonstrated. Therefore, we hypothesized that there would be fewer readmissions to inpatient care in the short-term course for the group that had been in FTA, but no difference in the long-term course of AN except better psychosocial adaption in the FTA group.

## Methods

### Participants

**Figure 1:**
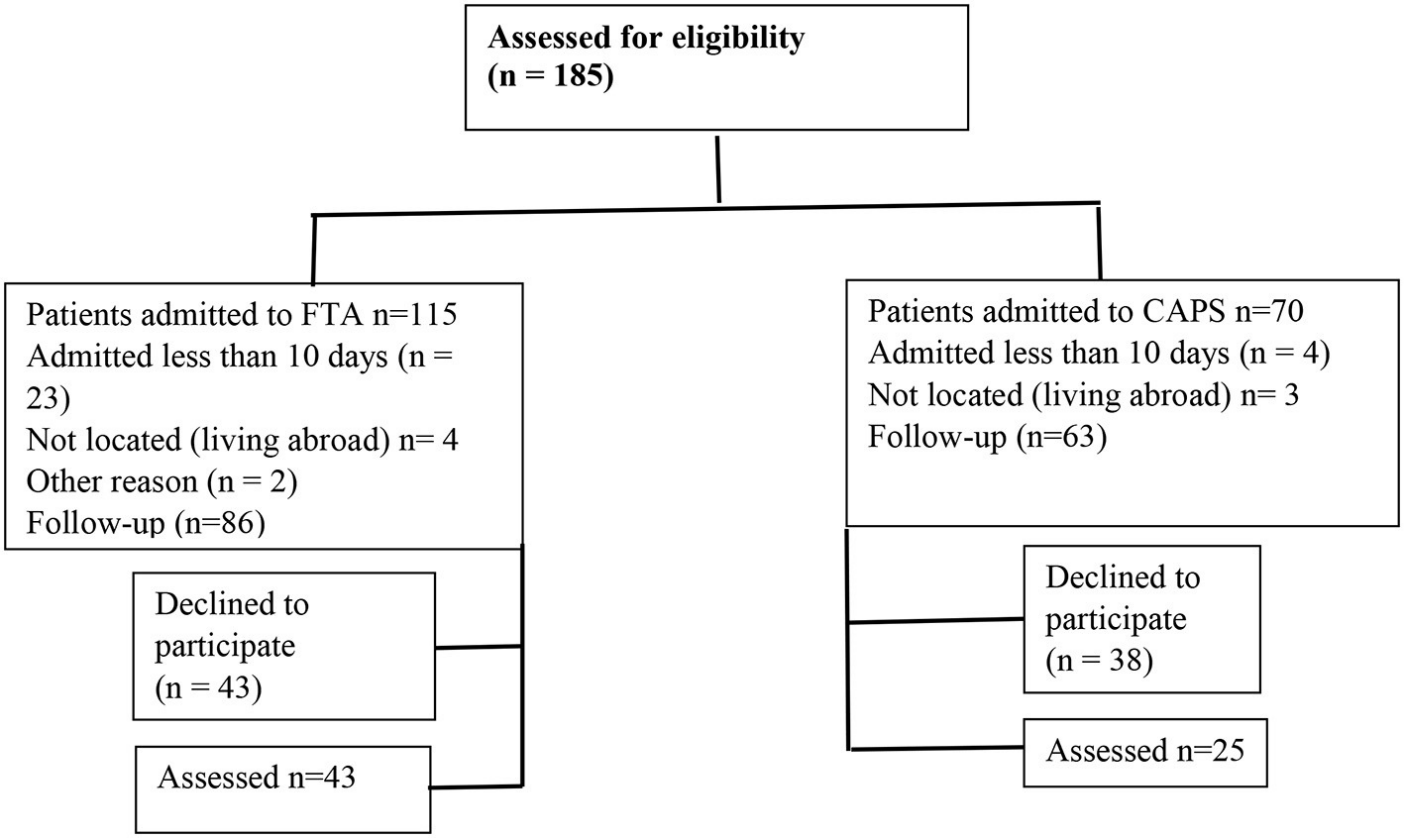
Flowchart.

During the period 1990–2009, 185 patients with the AN diagnosis were admitted to either inpatient care at CAMHS or FTA, 115 to FTA, and 70 to CAMHS. The participants selected for the follow-up were required to have been admitted for at least 10 days, as a shorter period may not be meaningful to evaluate. Eighty-six families admitted to the FTA, and 63 patients admitted to the CAMHS stayed there for a period longer than 10 days. These 149 former patients were invited to participate in a follow-up. Of those, 68 persons consented, 43 from the FTA group, 3 boys and 40 girls, and 25 from the CAMHS group, 2 boys and 23 girls, as shown in Figure 1. The mean age at admission was 14.8 years for the whole group.

### Treatment Programs

FTA was developed as an intensive family therapy alternative to psychiatric inpatient treatment specifically for AN (3). Patients and families are admitted to FTA when they are in a compromised medical state and with nationwide referrals. One family at a time lives in the apartment.

In the FTA model, the family is seen as a crucial resource in the process of the patient's recovery. The treatment focuses on strengthening the family's ability to challenge the AN during family meals, and therefore, the focus is on family meals as well as on family sessions—conjoint, separate, and individual. The focus of treatment in FTA is to strengthen parental cooperation and help them take responsibility for what the patient eats. The treatment also includes regular family therapy sessions, body awareness therapy, parental groups, and activities to normalize family life. The FTA is taking place in an ordinary apartment in a residential area in Lund. The families normally stay for approximately 5–8 weeks, of which a substantial part include home-leave to promote the transfer of acquired skills to the home setting. After discharge, the families are offered three follow-up sessions. Then, the local CAMHS takes responsibility for the treatment, and we do not know what treatment the patients receive.

The CAMHS is a traditional child psychiatric inpatient unit with a mix of diagnoses. The unit is not specialized in eating disorders (EDs). The patients are admitted mainly due to a compromised medical state, and all are drawn from the Malmö catchment area. One parent is encouraged to stay on the unit. Treatment focus is on weight gain, and most often, there is a weight goal for discharge. The treatment is not manualized, and no expected length of treatment has been formulated. The main intervention, stable over the years, is that the staff is responsible for the meals and that the parent can participate. After discharge, the local CAMHS reassumes responsibility for the treatment.

The main difference between the two treatment models is that the staff at the inpatient unit is responsible for the meal, whereas in FTA, the parents carry the meal responsibility, and the family lives in an ordinary housing apartment.

### Assessment at Follow-Up

The follow-up consisted of a personal meeting with two structured interviews, Structured Clinical Interview for Diagnostic, and Statistical Manual of Mental Disorders, 5th Edition (DSM-5), I and a semi-structured clinical interview about their life situation and state of health.

The Structured Clinical Interview for diagnosis (Structured Clinical Interview for DSM-5) (23) is a diagnostic interview based on DSM IV (24). When the follow-up interviews were conducted, there was no upgraded version in relation to DSM-5 in Swedish.

The semi-structured interview developed for the follow-up (unpublished manuscript in Swedish, available on request to the corresponding author) aimed to gather information about the participants' life situation, family, studies, work situation, and state of health, both in relation to the current situation as well as covering the follow-up period.

The following self-rating questionnaires were used:

- Eating Disorders Inventory, 3rd edition (25), assesses both eating disorder symptoms and psychological problems associated with an ED and consists of two scales, Eating Disorder Risk Composite and General Psychological Maladjustment Composite. It has been validated for use in Sweden (25).
- Symptom Check List (26) assesses general mental health.
- Eating Disorder Examination—Questionnaire assesses eating disorder symptoms. It has been validated for use in Sweden (27).
- Body Attitude Test (28) assesses body image disturbances and body dissatisfaction.
- RAND 36 is a public version of SF-36 that assesses the quality of life. It has been validated for use in Sweden (29).
- Morgan–Russel Outcome Assessment Schedule (30). A well-established outcome instrument in AN research. Subscale D on sexuality aspects was omitted.

At the follow-up, weight and height were measured.

### Statistical Analyses

Independent and paired *t*-tests were used to investigate differences between and within participants. All analyses were two-tailed. The alpha level was set at *p* ≤ 0.05. Chi-square tests were used when categorical data were analyzed.

## Results

At admission, there was no difference in age, comorbidity, or body mass index between the two groups, as shown in Table 1.

**Table 1:** Participant characteristics of the sample during treatment.

	**FTA**		**CAMHS**		***p***
	**Mean (SD)**	**Range**	**Mean ± SD**	**Range**	
*N*	43		25		
Age at onset (years)	13.4 (2.1)	7.0–16.0	14.0 (1.6)	11.4–18.0	0.207
Age admission (years)	14.5 (2.1)	9.5–17.4	15.1 (1.6)	11.8–18.5	0.285
%EBW at admission	76.8 (9.8)	58.0–106.2	76.4 (10.2)	59.8–98.2	0.829
%EBW discharge	80.8 (10.0)	57.4–104.3	88.1 (11.8)	61.7–105.2	0.013
Duration of admission (days)	42.1 (20.4)	7–91	75.7 (66.4)	8–231	0.007
Weight gain (kg/week)	0.29 (0.63)	−0.70–3.34	0.69 (0.53)	−0.06–2.24	0.011
Weight gain (%EBW/week)	0.71 (1.7)	−1.45–10.8	1.33 (1.0)	−0.12–4.4	0.128

*FTA, family treatment apartment; CAMHS, Child and Adolescent Mental Health Service; SD, standard deviation; %EBW percentage of expected weight*.

The treatment duration in FTA was shorter than at CAMHS, and the discharge weight was lower for the FTA group. Readmissions due to weight loss within 6 months from discharge were less common for FTA than CAMHS [two participants in the FTA group (4.7%) compared to eight participants in the CAMHS group (32.0%); *p* = 0.017]. Readmissions within 12 months were similar [eight participants in the FTA group (20.9%) compared to eight participants in the CAMHS group (32.0%) *p* = 0.174].

Half of the participants were readmitted at some point during the follow-up period, with no difference between the groups.

At admission, all participants were diagnosed as AN restrictive type. At follow-up, we re-diagnosed the FTA group but not the CAMHS group because the information in the patients' files was too scarce. We found that 4 of 43 in the FTA group fulfilled an avoidant/restrictive food intake disorder diagnosis at admission, two boys and two girls. At follow-up, one of the girls still fulfilled an avoidant/restrictive food intake disorder diagnosis, but the other three had no eating disorder. For all the participants, 32% had an eating disorder at follow-up, with no difference between the groups. The FTA group had fewer non-ED psychiatric diagnoses compared with the CAMHS group, but the difference was not significant (16.3 vs. 32.0% *p* = 0.132).

The follow-up took place on average 14.2 years after admission to treatment. The FTA group had a longer follow-up time than the CAMHS group, as shown in Table 2.

**Table 2:** Participant characteristics at follow-up.

**Follow-up**	**FTA**		**CAMHS**		***p***
***N***	**43**		**25**		
	**Mean ± SD**	**Range**	**Mean ± SD**	**Range**	
Age at follow-up (years)	30.1 (5.4)	19.0–39.0	27.6 (5.2)	19.1–38.0	0.073
Follow-up time (years)	15.5 (5.0)	6.1–24.8	12.6 (4.0)	6.9–21.5	0.021
BMI (kg/m^2^)	21.2 (3.3)	16.7–36.7	20.9 (3.3)	14.0–30.0	0.675
BAT	33.1 (24.6)	5–93	40.8 (18.8)	18–79	0.187
EDE-Q	1.30 (1.53)	0.0–4.9	1.59 (1.26)	0.0–4.3	0.442
EDI EDRC	110.6 (25.5)	84–187	115.7 (22.9)	91–165	0.412
EDI GPMC	366.5 (67.2)	253–509	424.6 (56.2)	333–557	0.001
SCL 90 GSI	0.54 (0.45)	0.0–1.7	0.89 (0.51)	0.15–1.94	0.005
MORGAN RUSSELL AO	9.8 (2.1)	4.4–12.0	8.6 (2.4)	3.2–12.0	0.027
RAND	585.0 (151.9)	240–783	484.3 (147.7)	271–733	0.012

*FTA, Family Treatment Apartment; CAMHS, Child and Adolescent Mental Health Service; SD, standard deviation; BMI, body mass index; BA, Body Attitude Test; EDE-Q, Eating Disorder Examination Questionnaire; EDI EDRC, Eating Disorders Inventory Eating Disorder Risk Composite; EDI GPMC, Eating Disorders Inventory General Psychological Maladjustment Composite*.

There was no difference in eating disorder pathology assessed by the Eating Disorder Examination Questionnaire and Eating Disorders Inventory Eating Disorder Risk Composite. There were no differences in body mass index.

According to Morgan–Russell Outcome Assessment Schedule, the FTA group had a better outcome on the average outcome score. The FTA group also had a better outcome on Symptom Checklist-90 and Eating Disorders Inventory General Psychological Maladjustment Composite, as shown in Table 2. The FTA group had a better quality of life score, as measured by RAND 36.

## Discussion

This study evaluates the long-term course of a group of patients who have been in family-based inpatient care and compares it with regular child psychiatric inpatient care. The general outcome is comparable between the groups and seems to be in line with what could be expected when compared with other studies. Full recovery in long-term follow-up studies of young people hospitalized varied between 41.5 and 75.8%. In our study, when we define full recovery as not fulfilling any eating disorder diagnosis or any other psychiatric diagnoses, 51.2% in the FTA group and 36.0% in the CAMHS group achieved full recovery. The FTA group seems to have a more favorable outcome. If the higher percentage of patients that met the criteria for a full recovery in the FTA group was due to family involvement or other factors is difficult to ascertain. The extent to which this relates to the initial eating disorder treatment, selection bias, or other factors is challenging to estimate. In this study, we were unable to gather information about treatment during the follow-up period, which also may have influenced the results.

During the first 6 months after discharge, the FTA group had fewer readmissions due to weight loss, despite having shorter admissions, and poorer weight gain at discharge. Discharge from FTA was motivated by medical stability and that the parents have control over what the patient eats so that the treatment could continue at home. Discharge from the inpatient unit was motivated by a predetermined weight gain that sometimes could take a long time to achieve. The longer stay at the inpatient unit did not yield a better prognosis, which has also been shown in previous research (31). This indicates that intensive treatment to enhance parental control may contribute to a stabilized weight gain in the first 6 months after discharge.

At follow-up, there was no difference in eating disorder pathology or eating disorder diagnosis between the groups. The FTA group had a better outcome in regard to general psychiatric pathology and a better quality of life. Although, it is difficult to make firm conclusions about the effect of treatment after such a long time, there may be some possible links. The higher quality of life score in the FTA group is in line with the study hypothesis. Even at times of severe illness, FTA may help sustain normal family life, which may protect the patient's social skills and consequently improve quality of life. Similar mechanisms may also influence the difference in general psychiatric pathology.

Further, research is needed to understand whether FTA is a non-inferior treatment compared with inpatient care. Compared with inpatient care, FTA offers high treatment intensity with shorter treatment duration, less staff involvement, and superior family involvement.

## Limitations

A major limitation of this study is that only 50% in the FTA group and 39.7% in the CAMHS group participated in the follow-up. A selection bias could influence the result. The lack of randomization also gives the possibility that the outcomes were influenced by selection bias. Specifically, in FTA, families had to agree to participate in treatment to be admitted, whereas, the inpatient group did not require family consent. Another possible bias or confounder may be geographical: FTA included patients from the entire country; the inpatient group had patients from the southern region of Sweden.

Another limitation is the long period during which the participant may have had different types of treatment that may impact the outcome. Another limitation is the differing follow-up time between the groups.

## Data Availability Statement

The original contributions presented in the study are included in the article/supplementary material, further inquiries can be directed to the corresponding author/s.

## Ethics Statement

The studies involving human participants were reviewed and approved by Regional Ethic Review Board, Lund University. The patients/participants provided their written informed consent to participate in this study.

## Author Contributions

UW is responsible for the study and RH is responsible for the follow-up interviews. Both authors contributed to the article and approved the submitted version.

## Conflict of Interest

The authors declare that the research was conducted in the absence of any commercial or financial relationships that could be construed as a potential conflict of interest.
